# Supplementary material for: The genetic mechanism of B chromosome drive in rye illuminated by chromosome-scale assembly
Source: Nat Commun. 2024 Nov 8;15:9686. doi: 10.1038/s41467-024-53799-w (PMC11549084; doi:10.1038/s41467-024-53799-w)
Supplement: Supplementary file 1 — Supplementary information [file 41467_2024_53799_MOESM1_ESM.pdf]

The genetic mechanism of B chromosome drive in rye illuminated by chromosome-scale assembly

Chen *et al.*

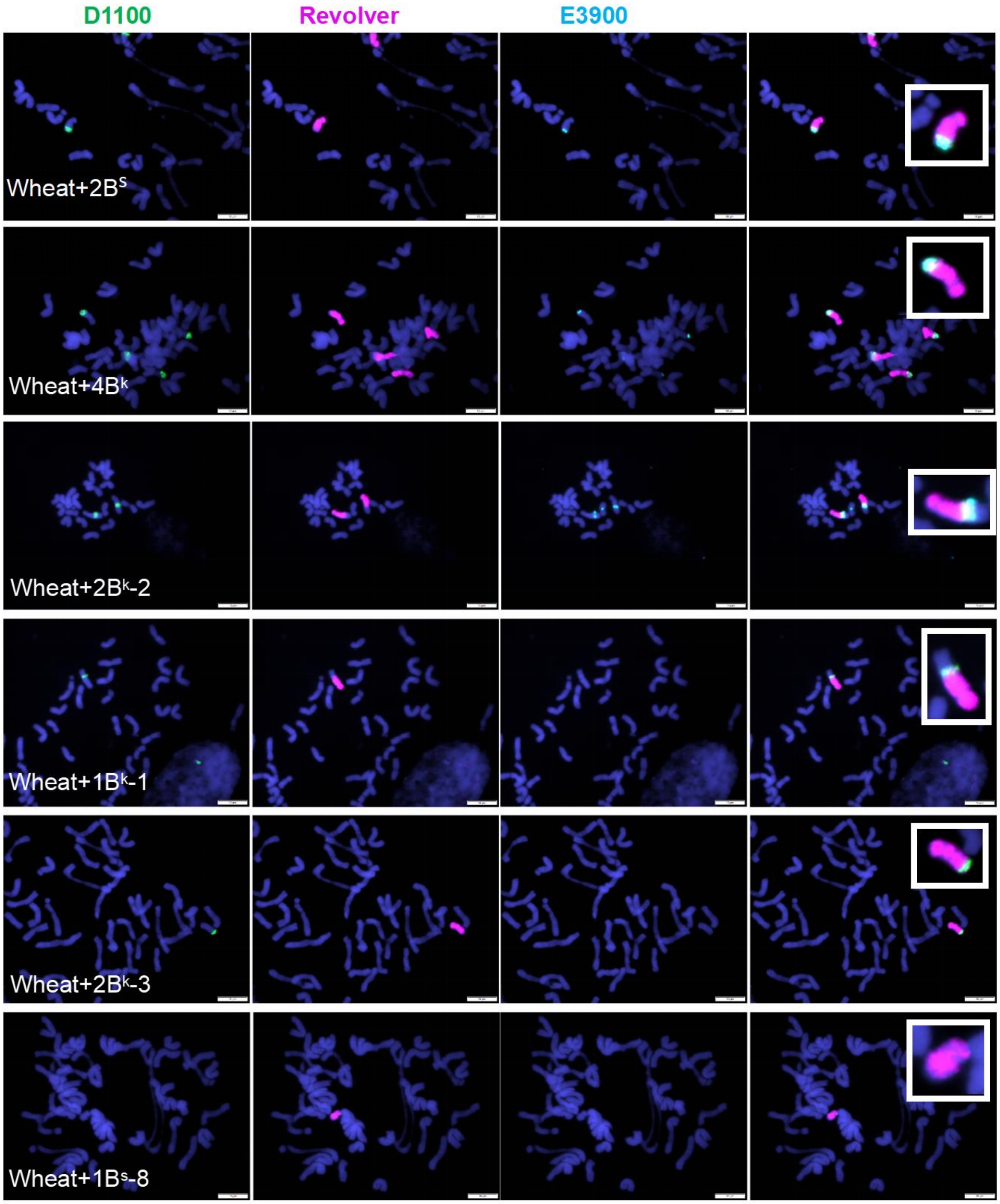

Supplementary Fig. 1. Characterization of rye B chromosome variants (B^s-8, B^s, B^k, B^k-1, B^k-2, B^k-3) in the background of wheat by FISH. Mitotic metaphase chromosomes were labelled with D1100 (green), Revolver (magenta) and E3900 (sky blue); Chromosomes were counterstained with DAPI (blue). Insets showing selected, further enlarged B variants. Bar =10 µm.

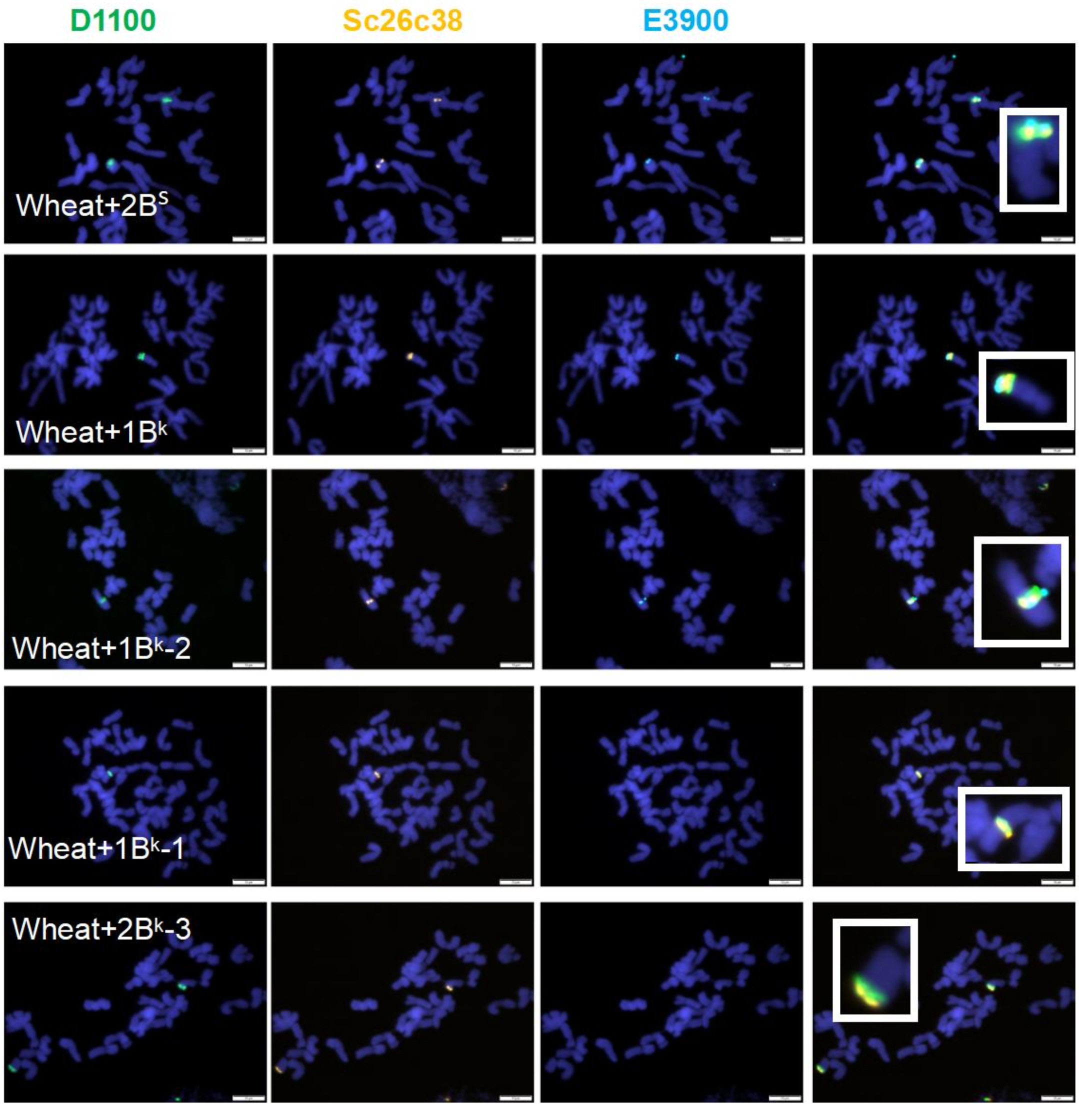

Supplementary Fig. 2. Characterization of rye B chromosome variants (B^s, B^k, B^k-1, B^k-2, B^k-3) in the background of wheat by FISH. Mitotic metaphase chromosomes were labelled with D1100 (green), Sc26c38 (orange) and E3900 (sky blue); Chromosomes were counterstained with DAPI (blue). Insets showing selected, further enlarged B variants. Bar =10 µm.

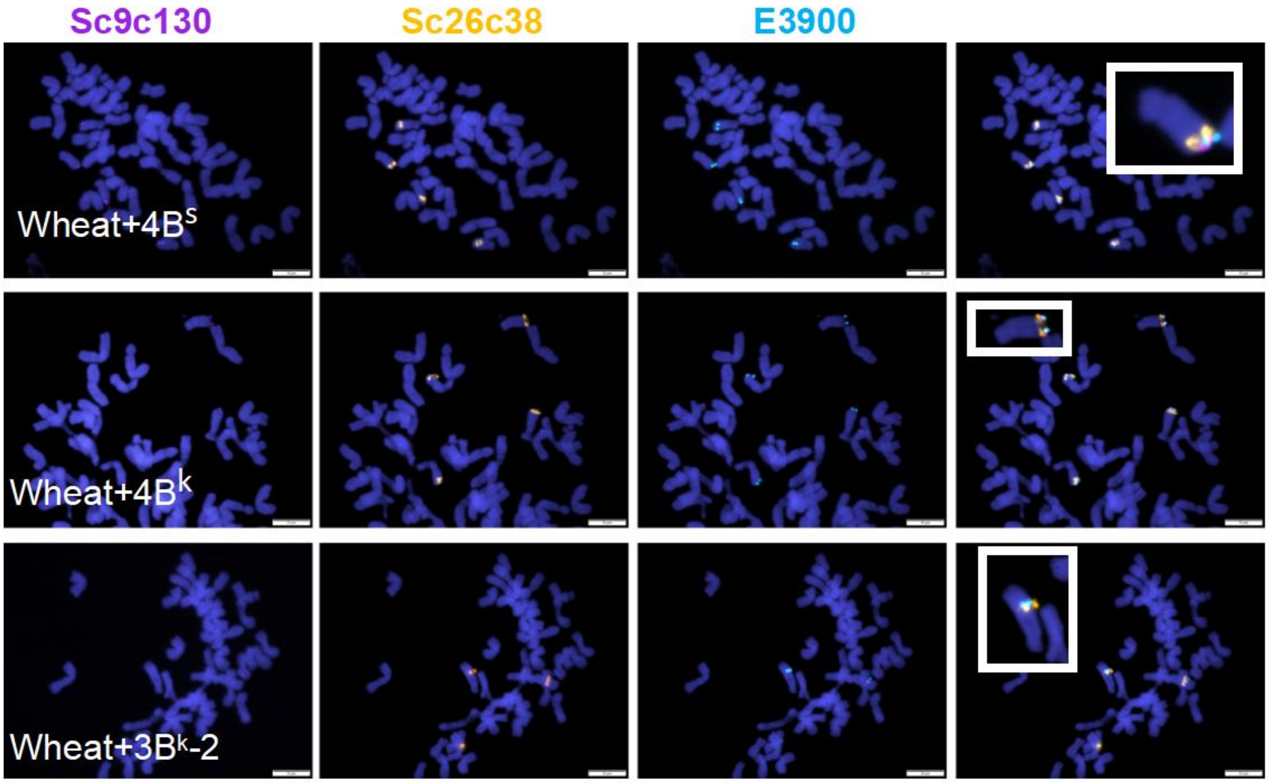

Supplementary Fig. 3. Characterization of rye B chromosome variants (B^s, B^k, B^k-2) in the background of wheat by FISH. Mitotic metaphase chromosomes were labelled with Sc9c130 (violet), Sc26c38 (orange), E3900 (sky blue). Chromosomes were counterstained with DAPI (blue). Insets showing selected, further enlarged B variants. Bar =10 µm.

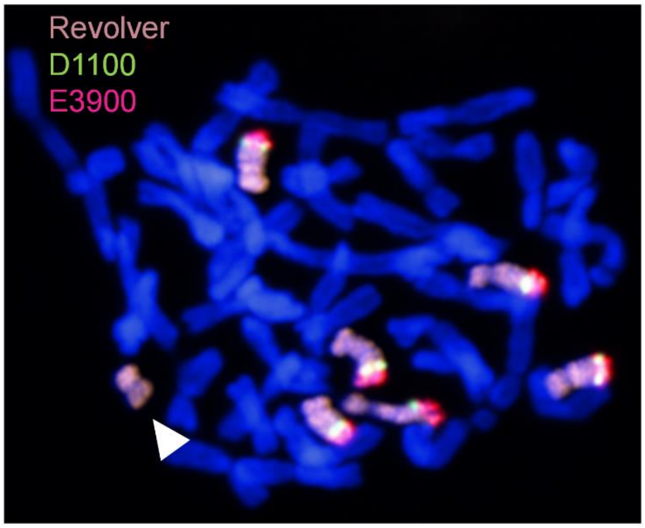

Supplementary Fig. 4. A mitotic cell of wheat cv. Chinese Spring with six rye standard B^s and a truncated B-fragment (arrow) after FISH. FISH was performed using the B-specific repeats D1100 (green) and E3900 (magenta) and the rye genome-specific repeat Revolver (pink) as probes.

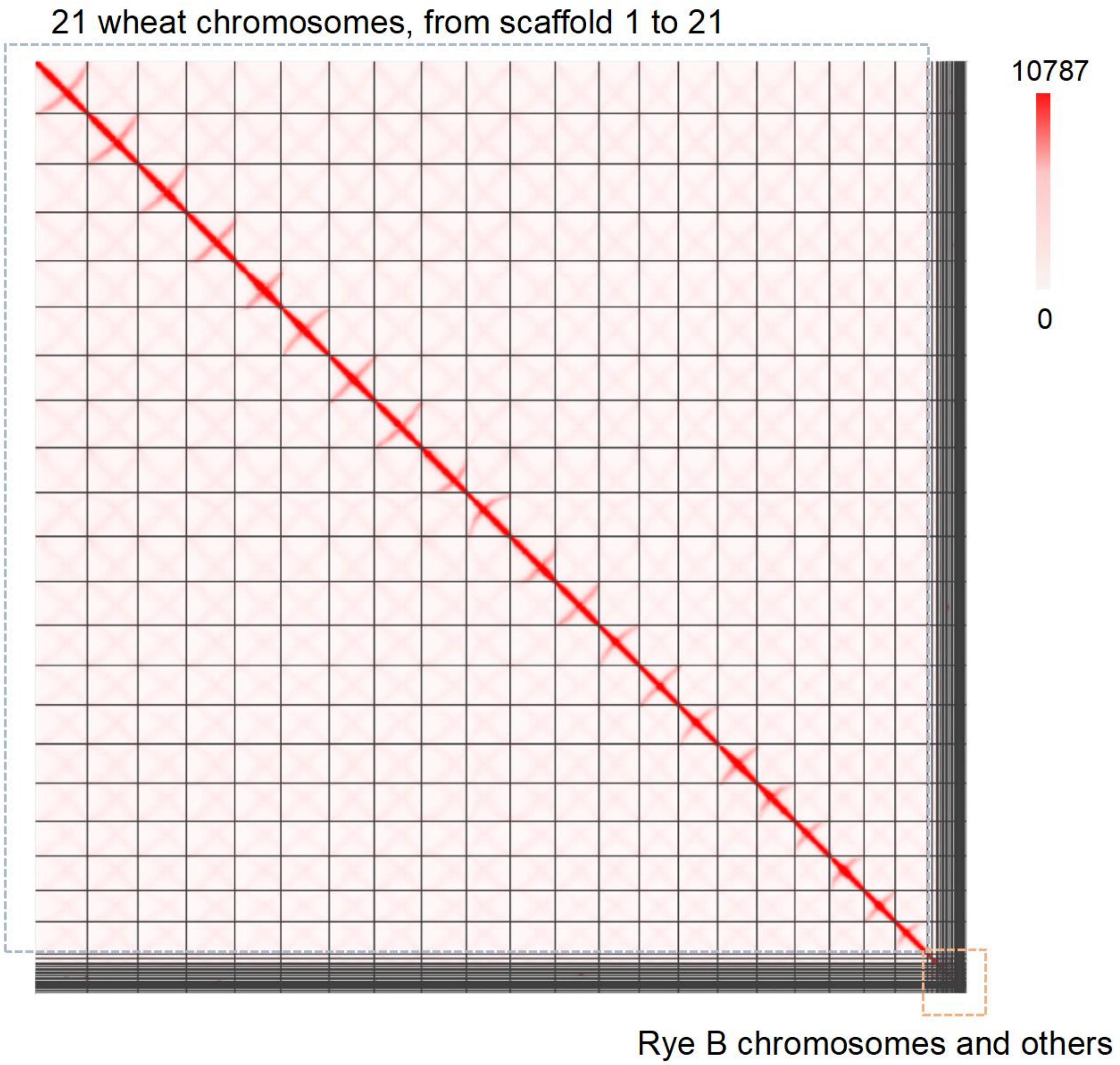

Supplementary Fig. 5. Hi-C scaffolds the 21 wheat chromosomes but fails to scaffold the rye B chromosome. Hi-C interaction heatmap of the scaffolds of wheat with the rye B chromosomes. Color bar on the right represents the density of Hi-C interactions, which are indicated by the number of links at the 5-Mb resolution. The 21 large scaffolds that show Rabl configuration come from the wheat genome, and the remaining small scaffolds represent the rye B chromosome and unscaffolded wheat contigs.

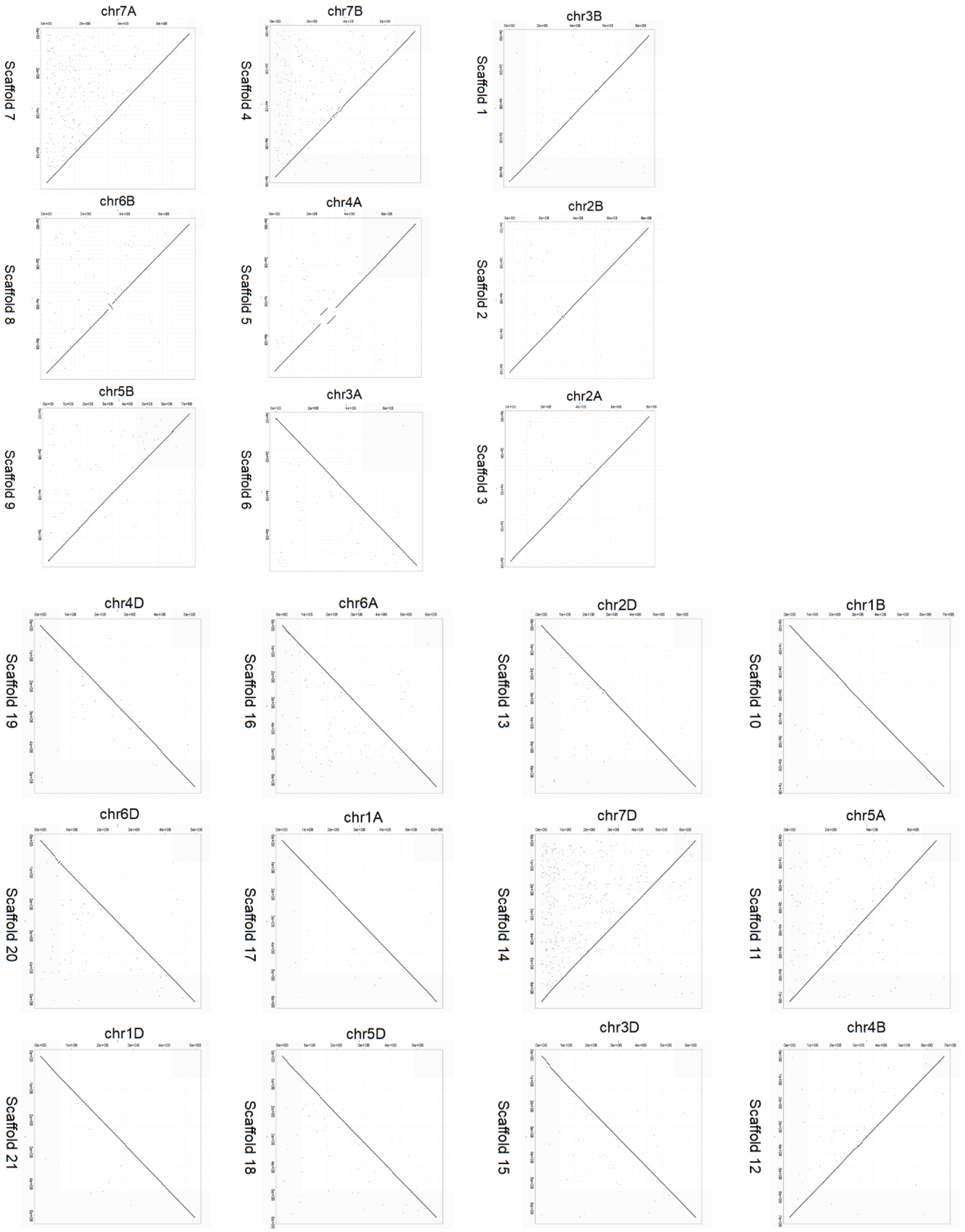

Supplementary Fig. 6. Alignment of the 21 large scaffolds to the wheat genome reveals their corresponding chromosomes. Sequence alignment between scaffold (y-axis) and chromosome wheat (x-axis).

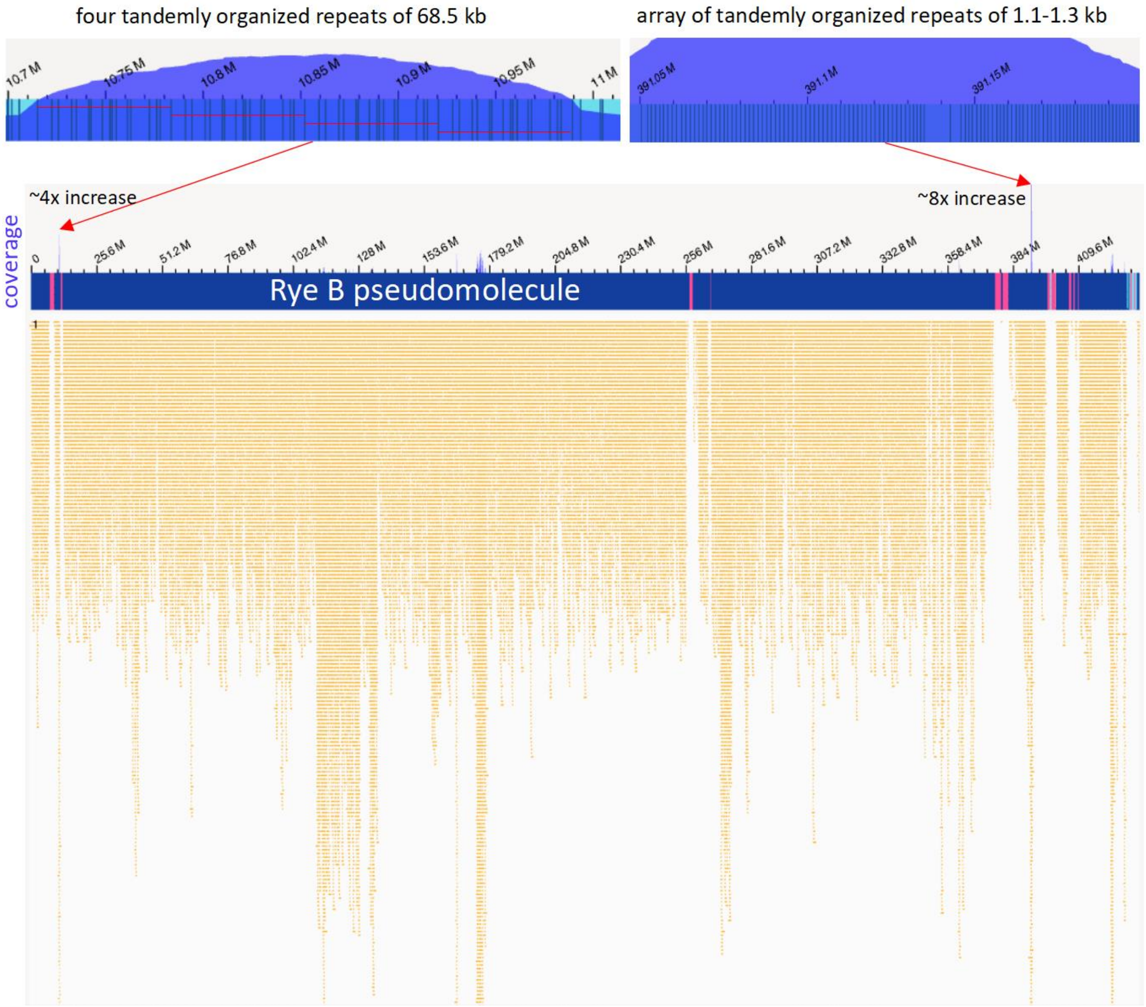

Supplementary Fig. 7. Putative collapsed regions in rye B pseudomolecule. Labelled DNA molecules (yellow lines) that were used to generate the rye B optical genome map were aligned to the rye B pseudomolecule (blue bar). The narrow violet peaks above the bar indicate regions with multiple increases in molecule coverage, suggesting a local sequence collapsing in the chromosome assembly. Two regions with the highest molecule coverage (red arrows), shown in the close-ups (top), are composed of tandemly organized repeats, which tend to collapse in sequence assemblies.

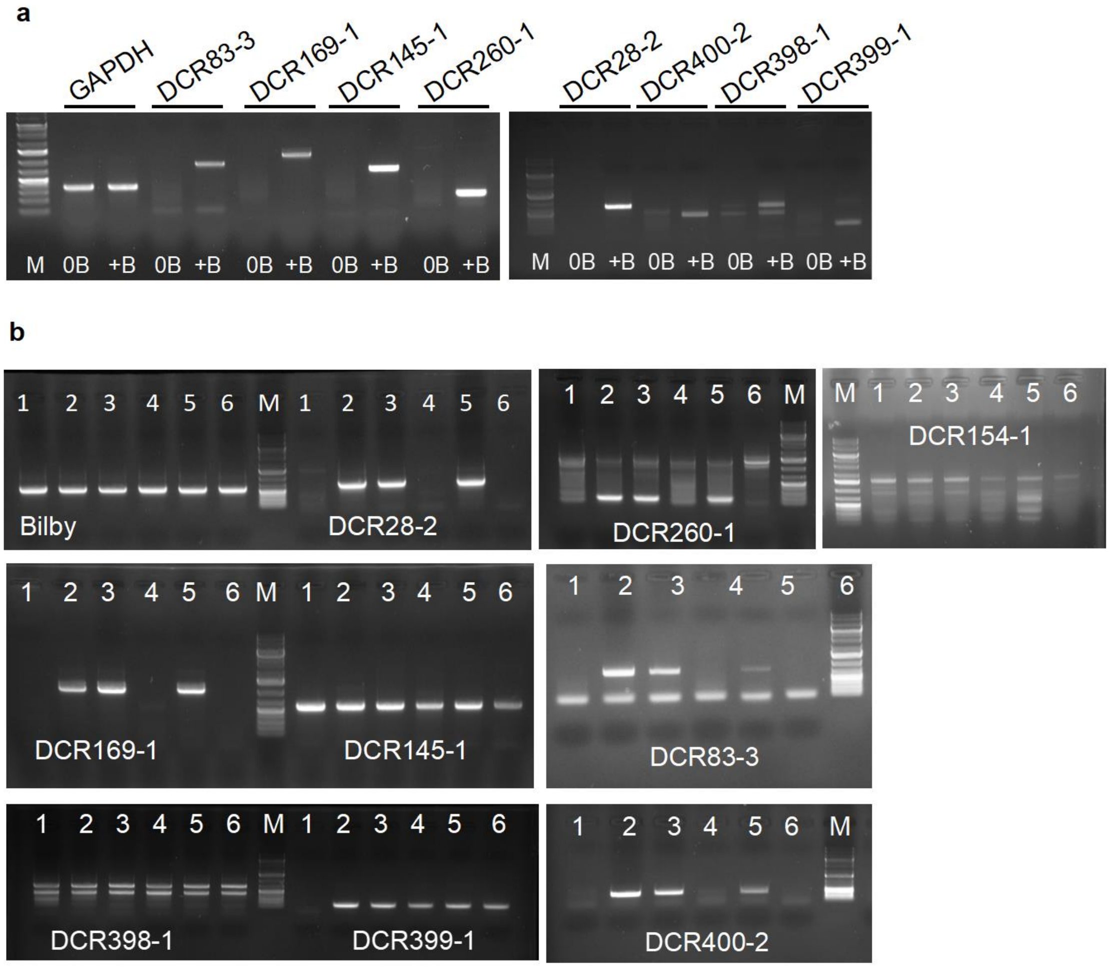

Supplementary Fig. 8. Genomic PCR to test the rye B drive control region-specific location of preselected candidates. (a) Testing B-specific primers in wheat with (+B) and without B chromosomes (0B). (M) Marker: 1kb plus DNA marker. GAPDH is a control primer for DNA quality; **(b)** Genomic DNA of wheat with drive-positive B variants ($2B^s$, $2B^k$, $1B^k$-2) or drive-negative B variants ($1B^s$-8, $1B^k$-1, $2B^k$-3) was used as a PCR template in combination with DCR-specific primers (Supplementary Table 6, 8). Lanes: wheat cv. Chinese Spring (1) $+B^s$-8; (2) $+2B^s$; (3) $+2B^k$; (4) $+1B^k$-1; (5) $+1B^k$-2; (6) $+2B^k$-3, (M) Marker: 1kb plus DNA marker. Source data are provided as a Source Data file.

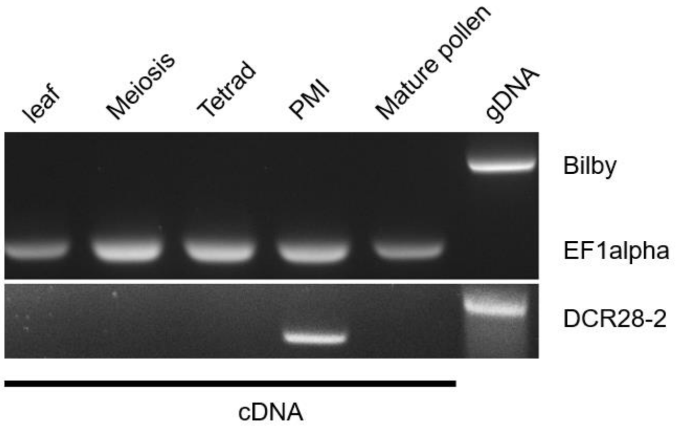

Supplementary Fig. 9. Reverse transcription polymerase chain reaction (RT-PCR) confirmed *DCR28* expresses in a tissue-specific way. cDNAs of wheat with $2B^s$ include leaf tissues, spikes undergoing meiosis, spikes at the tetrad stage, anthers undergoing the first pollen mitosis (PMI), and mature pollen. The quality of the cDNA was tested via control primer EF1alpha. Primer DCR28-2 amplified a smaller product in the cDNA than gDNA, indicating that there was no gDNA contamination in the cDNA templates. The quality of gDNA of wheat with $2B^s$ was tested by control primer Bilby. Source data are provided as a Source Data file.

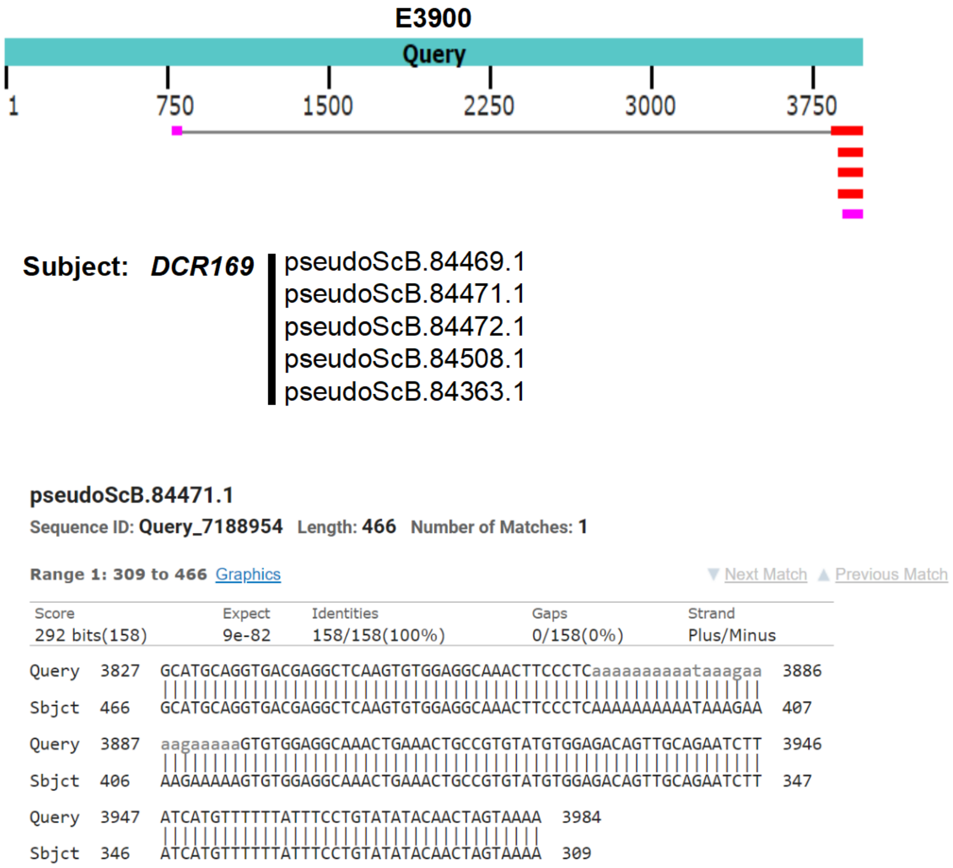

Supplementary Fig. 10. Blast alignment reveals that *DCR169* shares similarities with the end of the E3900 repeat. Query: E3900, subject: 5 genes of *DCR169* (pseudoScB.84469.1, pseudoScB.84471.1, pseudoScB.84472.1, pseudoScB.84508.1, pseudoScB.84363.1). The base pair alignment is shown between E3900 (GenBank: AF222021.1) and pseudoScB.84471.1.

Query: DCR28 of *Ae. speltoides* B chromosome (AesDCR28)
Subject: DCR28 of rye B chromosome

Score	Expect	Method	Identities	Positives	Gaps
213 bits(542)	7e-73	Compositional matrix adjust.	142/265(54%)	161/265(60%)	55/265(20%)

```
Query  16   PQPRPRRVFGTVRSSNPLAEKPARPPPQEHSKKLSPQPPPIPPIRAKAARLSRPAEKPLI  75
            PQPRPRR  GTVRSS  LA KPA PPPQ+HSK   P                 P  KPL
Sbjct  48   PQPRPRRALGTVRSSISLAWKPAPPPPQKHSKLPHP-----------------PRWKPLK  90

Query  76   KSCPAPPDLAAKAARPSRPAEKPLIKSPPDLAAKDRKKGQRVSLQGDVAALAAPGSGEKV  135
             S P P    AKAARPSRP  KPL K+ P  A  D               LAAP     V
Sbjct  91   VSPPIP----AKAARPSRPVVKPLKKACP--ATVD---------------LAAP-----V  124

Query  136  KTSTEDSDSDSGGRTPVLPVKALVVAAETPFFTAQKNKNGSRCTIDQLESASYWLAQIYL  195
            K S ++S     GRTP++ VKA     E  FFTAQ   + S  T++ LE ASYWLAQI+L
Sbjct  125  KPSAQNS----AGRTPMVHVKA---PEEDLFFTAQ---DWSSHTLNSLERASYWLAQIHL  174

Query  196  AESVGKHRVSAEFFRLAFECQAQPFQMIQSELENYAVRHESAIGSTLTALFPDLLVANGM  255
            +ES G H VSA+FF LAFECQAQP   I++EL NY VR+E+A  STLT LF +LLVA+ M
Sbjct  175  SESAGWHSVSAKFFGLAFECQAQPIHRIRTELRNYVVRYENA--STLTPLFRELLVAHAM  232

Query  256  AVNQLKFDTDGSEKVDTPTTTNTFD  280
             VN LKFDTDGSE+VDTPTTTNT D
Sbjct  233  PVNHLKFDTDGSEQVDTPTTTNTVD  257
```

Supplementary Fig. 11. Identification of DCR28-like gene on the B chromosome of *Aegilops speltoides* (AesDCR28). blastp alignment between DCR28 of *Ae.speltoides* B chromosome and DCR28 of rye B chromosome. The sequence of the *de novo* assembled transcript and its translated protein of AesDCR28 was provided in Supplementary Data 10.

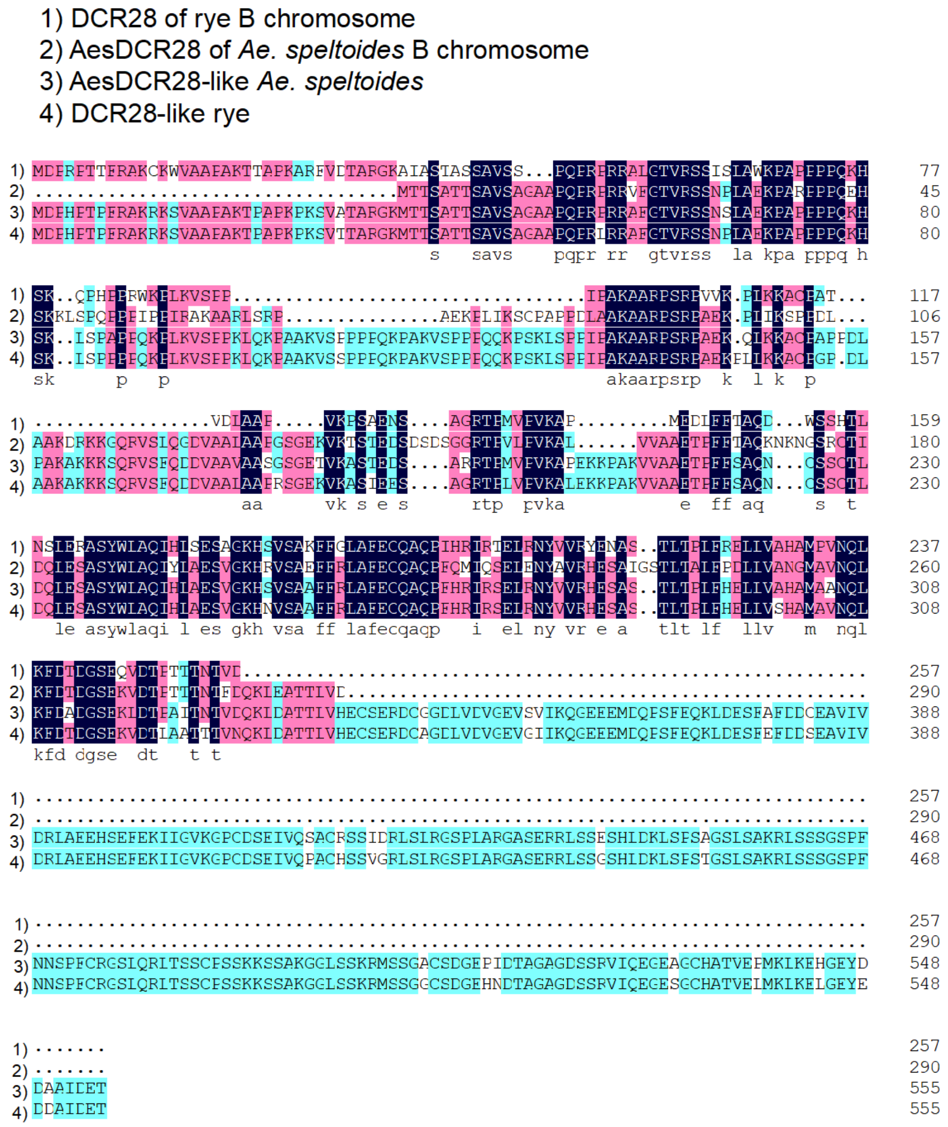

Supplementary Fig. 12. Multiple protein sequence alignment of the DCR28 of rye B chromosome, AesDCR28 of *Ae. speltoides* B chromosome and their paralogous proteins on the A chromosomes. Source data are provided as a Source Data file.

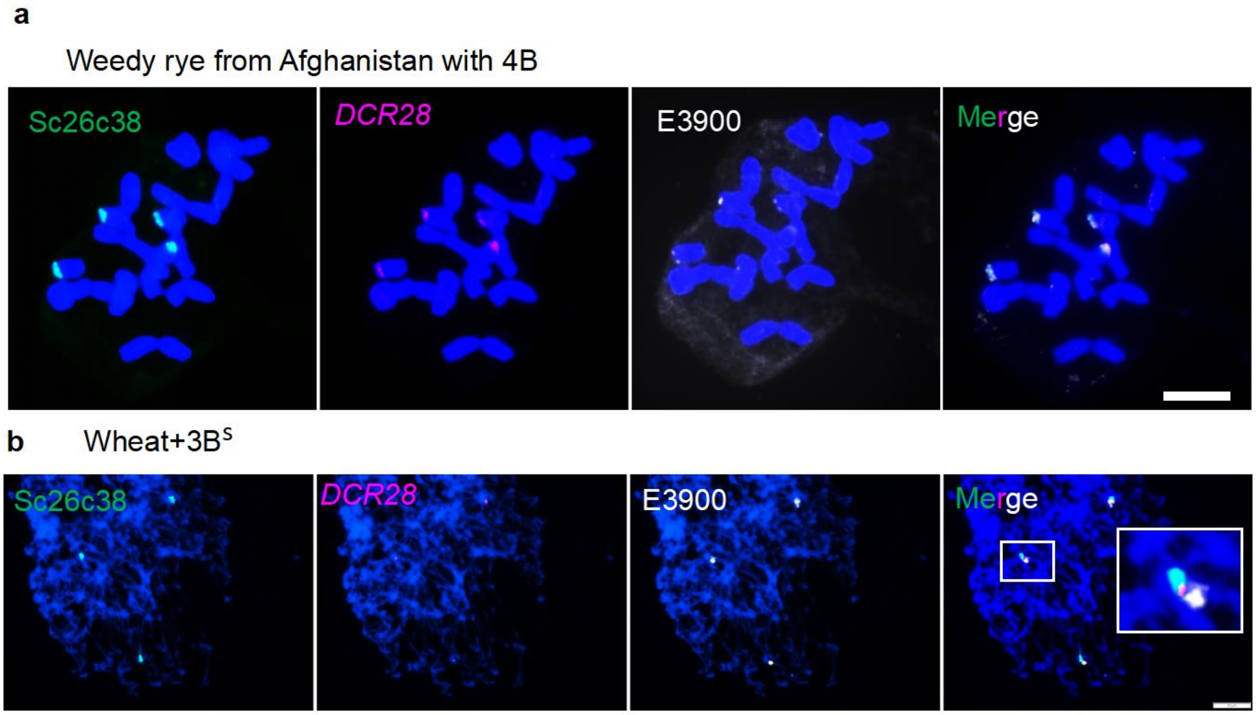

Supplementary Fig. 13. Rye B drive control region-specific position of DCR28 confirmed by FISH. (a) Mitotic metaphase of weedy rye from Afghanistan with 4B after FISH using Sc26c38 (green), *DCR28* (magenta) and E3900 (white) specific probes. **(b)** Pachytene chromosomes of wheat with $3B^s$ after FISH using Sc26c38 (green), *DCR28* (magenta) and E3900 (white) specific probes. DAPI was used to counterstain the chromosomes (blue), The inset shows selected, further enlarged Sc26c38-, *DCR28*- and E3900- signals. Bars = 10 µm.

```
pseudoScB.83929_len_260   MDPRPTTFR.......APAKTTAPKARFVDTARGKAAAP.......SPLPSHGRAARWAPS......GAASPWPQKHSMLPHPPRWKPLKVSPPIPAKAA    80
pseudoScB.83934_len_257   MDPRPTTFRAKCKWVAAPAKTTAPKARFVDTARGKAIASTASSAVSSPQPRPRRALGTVRSSISL.WKPAPPPPQKHSKLPHPPRWKPLKVSPPIPAKAA    99
pseudoScB.83936_len_298   MDPRPTTFRAKCKWVAAPAKTTEPKARFVDTARGKAIASTASSAVSSPQPRPRRALGTVRSSISLAWKPARPPPQKHSKQPHQPRWKPLKVSPPIPAKAA   100
pseudoScB.83937_len_258   MDPRPTTFRAKCKWVAAPAKTTAPKARFVDTARGKAIASTASSAVSSPQPRPRRALGTVRSSISLAWKPAPPPPQKHSKLPHPPRWKPLKVSPPIPAKAA   100
pseudoScB.83940_len_298   MDPRPTTFRAKCKWVAAPAKTTAPKARFVDTARGKAIASTASSAVSSPQPRPRRALGTVRSSISLAWKPAPPPPQKHSKQPHPPRWKPLKVSPPIPAKAA   100
pseudoScB.83941_len_298   MDPRPTTFRAKCKWVAAPAKTTAPKARFVDTARGKAIASTASRAVSSPQPRPRRALGTVRSSISLAWKPAPPPPQKHSKQPHQPRWKPLKVSPPIPAKAA   100
pseudoScB.83947_len_303   MDPRPTTFRAKCKWVAAPAKTTAPKARFVDTARGKAIASTASSAVSSPQPRPRRALGTVRSSISLAWKPAPPPPQKHSKLPHPPRWKPLKVSPPIPAKAA   100
pseudoScB.83948_len_258   MDPRPTTFRAKCKWVAAPAKTTAPKARFVDTARGKAIASTASSAVSSPQPRPRRALGTVRSSISLAWKPAPPPPQKHSKLPHPPRWKPLKVSPPIPAKAA   100
Consensus                 mdprpttfr       apaktt pkarfvdtargka a        sp p   ra     s        a p pqkhs  ph prwkplkvsppipakaa

pseudoScB.83929_len_260   RPSRPVPVVKPLKKACPATVDLAAPVKPSAENSAGRTPMVPVKAPAEDLFFTAQDWSSHTLNSLERASYWLAQIHLSESAGKHSVSAKFFRLAFECQAQP   180
pseudoScB.83934_len_257   RPSR..PVVKPLKKACPVTVDLAAPVKPSAENSAGRTPMVHVKAPEEDLFFTAQDWSSHTLNSLERASYWLAQIHLSESAGKHSVSAKFFGLAFECQAQP   197
pseudoScB.83936_len_298   RPSR..PVVKPLKKACPATVDLAAPVKPSAENSAGRTPMVPVKAPMEDLFFTAQDWSSHTLNSLERASYWLAQIHLSESAGKHSVSAKFFGLAFECQAQP   198
pseudoScB.83937_len_258   RPSR..PVVKPLKKACPATVDLAAPVKPSAQNSAGRTPMVHVKAPEEDLFFTAQDWSSHTLNSLERASYWLAQIHLSESAGWHSVSAKFFGLAFECQAQP   198
pseudoScB.83940_len_298   RPSR..PVVKPLKKACPATVDLAAPVKPSAENSAGRTPMVPVKAPMEDLFFTAQDWSSHTLNSLERASYWLAQIHLSESAGKHSVSAKFFGLAFECQAQP   198
pseudoScB.83941_len_298   RPSR..PVVKPLKKACPATVDLAAPVKPSAENSAGRTPMVPVKAPMEDLFFTAQDWSSHTLNSLERASYWLAQIHLSESAGKHSVSAKFFGLAFECQAQP   198
pseudoScB.83947_len_303   RPSR..PVVKPLKKACPATVDLAAPVKPSAQNSAGRTPMVHVKAPEEDLFFTAQDWSSHTLNSLERASYWLAQIHLSESAGKHSVSAKFFGLAFECQAQP   198
pseudoScB.83948_len_258   RPSR..PVVKPLKKACPATVDLAAPVKPSAQNSAGRTPMVHVKAPEEDLFFTAQDWSSHTLNSLERASYWLAQIHLSESAGKHSVSAKFFGLAFECQAQP   198
Consensus                 rpsr  pvvkplkkacp tvdlaapvkpsa nsagrtpmv vkap edlfftaqdwsshtlnslerasywlaqihlsesag hsvsakff lafecqaqp

pseudoScB.83929_len_260   IHRIRTELRNYVVRYENASTLTPLFRELLVANAMPVNQLKFDTDGSEQVDTPTTTNTV   238
pseudoScB.83934_len_257   IHRIRTELRNYVVRYENASTLTPVFRELLVAHAMPVNHLKFDTDGSEQVDTPTTTNTV   255
pseudoScB.83936_len_298   IHRIRTELRNYVVRYENASTLTPLFRELLVAHAMPVNQLKFDTDGSEQVDTPTTTNTV   256
pseudoScB.83937_len_258   IHRIRTELRNYVVRYENASTLTPLFRELLVAHAMPVNHLKFDTDGSEQVDTPTTTNTV   256
pseudoScB.83940_len_298   IHRIRTELRNYVVRYENASTLTPLFRELLVAHAMPVNQLKFDTDGSEQVDTPTTTNTV   256
pseudoScB.83941_len_298   IHRIRTELRNYVVRYENASTLTPLFRELLVAHAMPVNQLKFDTDGSEQVDTPTTTNTV   256
pseudoScB.83947_len_303   IHRIRTELRNYVVRYENASTLTPLFRELLVAHAMPVNHLKFDTDGSEQVDTPTTTNTV   256
pseudoScB.83948_len_258   IHRIRTELRNYVVRYENASTLTPLFRELLVAHAMPVNHLKFDTDGSEQVDTPTTTNTV   256
Consensus                 ihrirtelrnyvvryenastltp frellva ampvn lkfdtdgseqvdtptttntv
```

Supplementary Fig. 14. Multiple amino acid sequence alignment of eight DCR28 copies reveals they encode a conserved 256-aa protein. Source data are provided as a Source Data file.

```
DCR28                  MDPRPTTFRAKCKWVAAPAKTTAPKARFVD..TARGKAIASTASSAVSS...PQRPRRALGTVRSSISLAWKPAPPPPQ    75
DCR28-like_rye         MDPHPTPFRAKRKSVAAPAKTPAPKPKSVT..TARGKMTTSATTSAVSAGAAPQRPLRRAFGTVRSSNPLAEKPAPPPPQ    78
DCR28-like_wheat_B     MDPHPTPFRAKRKSVAAPAKTPAPKPKSVA..TARGKMTTSATTSAVSAGAAPQRPPRRAFGTVRSSNSLAEKPAPPPPQ    78
DCR28-like_wheat_D     MDPHPTPFRAKRKSVAAPAKTPAPKPKSVT..TARGKMTTSATTSAVSGGAAPQRPPRRAFGTVRSSNPLAEKPAPPPPQ    78
DCR28-like_wheat_A     MDPHPTPFRAKRKSVAAPAKTPAPKPKSVANGTARGRMTTSATTSAVSAGAAPQRPPRRAFGTVRSSNSLAEKPAPPPPQ    80
Consensus              mdp pt frak k vaapakt apk   v  targ    s   savs    pqpr rra gtvrss  la kpappppq

DCR28                  KHSKLPHPPRWKPLKVSPP..................................IPAKAARPSRPVVKP.LKKACPATVDL   120
DCR28-like_rye         KHSKLSPPPPQKPLKVSPPKLQKPAAKVSSPPPQKPAKVSPPPQQKPSKLSPPIPAKAARPSRPAEKPLLKKACPGP.DL   157
DCR28-like_wheat_B     KHSKLSPPPPQKPLKVSPPKLQKPAAKVSPPPPQKPAKVSPPPQQKPSKLSPPIPAKAARPSRPAEKP.LKKACPAPPDL   157
DCR28-like_wheat_D     KHSKLSPPPPQKPLKVSPPKLQKPAAKLSPPPPQKPAKVSPPPQQKPSKLSPPIPAKAARPSRPAEKP.LKKACPAPPDL   157
DCR28-like_wheat_A     KHSKLSPPPPQKPLKVSPPKLQKPAAKVSPPPPQKPAKVSPPPQQKLSRLSPPVPAKAARPSRPAEKP.LKKACPAPPDL   159
Consensus              khskl  pp  kplkvspp                                  pakaarpsrp  kp lkkacp   dl

DCR28                  AAPVKPSAQ.........................NSAGRTPMVHVKAPE.........EDLFFTAQDWSSHTLNSLERAS   166
DCR28-like_rye         AAKAKKKSQRVSFQDDVAALAAPRSGEKVKASIEESAGRTPLVPVKALEKKPAKVVAAETPFFSAQNCSSCTLDQLESAS   237
DCR28-like_wheat_B     AAKAKKKSQRVSFQDDVAAVAASGSGEKVKASTEDSAGRTPMVPVKALEKKPAKVVVAETPFFSAQNCSSCTLDQLESAS   237
DCR28-like_wheat_D     AAKAKKKSQRVSFQDDVAALAVPGSGEKVKA.TEDSAGRTPMVPVKALEKKPAKVVAAETPFFSAQNCSSCTLDQLESAS   236
DCR28-like_wheat_A     AAKAKKKSQRVSFQDDVAALAPSGSGEKVKASTEDSAGRTPMVPVKPLEKKPAKVVAAETPFFSAQNCSSCTLDQLESAS   239
Consensus              aa  k   q                          sagrtp v vk  e         e  ff aq   ss tl  le as

DCR28                  YWLAQIHLSESAGKHSVSAKFFGLAFECQAQPIHRIRTELRNYVVRYENASTLTPLFRELLVAHAMPVNHLKFDTDGSEQ   246
DCR28-like_rye         YWLAQIHLAESVGKHNVSAAFFRLAFECQAQPFHRIRSELRNYVVRHESASTLTPLFHELLVSHAMAVNQLKFDTDGSEK   317
DCR28-like_wheat_B     YWLAQIHLAESVGKHNVSAAFFRLAFECQAQPFHRIRSELRNYVVRHESASTLTPLFHELLVAHAMAANQLKFDTDGSEK   317
DCR28-like_wheat_D     YWLAQIHLAESVGKHNVSAAFFRLAFECQAQPFHRIRSELRNYVVRHESASTLTPLFHELLVAHAMAVNQLKFDTDGSER   316
DCR28-like_wheat_A     YWLAQIHLAESVGKHNVSAAFFRLAFECQAQPFHRIRSELRNYVARHESASTLTPLFHELLVAHAMAVNQLKFDTDGSEK   319
Consensus              ywlaqihl es gkh vsa ff lafecqaqp hrir elrnyv r e astltplf ellv ham  n lkfdtdgse

DCR28                  VDTPTTTNTVD.....................................................................   257
DCR28-like_rye         VDTLAATTTVNQKLDATTLVHECSERDCAGDLVDVGEVGIIKQGEEEMDQPSFEQKLDESFEFDDSEAVIVDRLAEEHSE   397
DCR28-like_wheat_B     VDTPATTNTVDQKLDATTLVHECSERDCGGDLVDVGEVSVIKQGEEEMDQPSFEQKLDESFAFDDCEAVIVDRLAEEHSE   397
DCR28-like_wheat_D     VDTPATTNTVDQKLDATTLVHECSERDCGGDLVDVGEVSVIKQGEEEMDQPSFEQKLDASFAFDDCEAVIVDRLAEEHSE   396
DCR28-like_wheat_A     VDTPATTNTVDQKLDATTLVHECSERDCGGDLVDVGEVSVIKQGEEEMDQPSFEQKLDESFAFDDCEAVIVDRLAEEHSE   399
Consensus              vdt   t tv

DCR28                  ................................................................................   257
DCR28-like_rye         FEKIIGVKGPCDSEIVQPACHSSVGRLSLRGSPLARGASERRLSSGSHLDKLSPSTG.SLSAKRLSSSGSPFNNSPFCRG   476
DCR28-like_wheat_B     FEKIIGVKGPCDSEIVQSACRSSIDRLSLRGSPLARGASERRLSSESHLDKLSPSAG.SLSAKRLSSSGSPFNNSPFCRG   476
DCR28-like_wheat_D     FEKIIGVKGPCDSETVQSACRSSIDRLSLRGPPLARGASERRLSSESHLDKLSPSAG.SLSAKRSSSSGSPFNNSPFCRG   475
DCR28-like_wheat_A     FEKIIGVKGPCDSEIVQSACRSSIDRLSLRGSPLARGASERRLSSESHLDKLSPSAAGSLSAKRLSSSGSPFNNSPFCRG   479
Consensus

DCR28                  ................................................................................   257
DCR28-like_rye         SLQRLTSSCPSSKKSSAKGGLSSKRMSSGGCSDGEHN.DTAGAGDSSRVIQEGESGCHAT..........VELMKLKELG   545
DCR28-like_wheat_B     SLQRLTSSCPSSKKSSAKGGLSSKRMSSGACSDGEPI.DTAGAGDSSRVIQEGEAGCHAT..........VEPMKLKEHG   545
DCR28-like_wheat_D     SLQRLTSSCPSSKKSSAKGGLSSKRMSSGGCSDGEPI.DTAGAGDSSRVIEEGEAGCHATGMLTCAELHAVEPMKLKEHG   554
DCR28-like_wheat_A     SLQRLTSSCPSSKKSSAKGGLSAKRMSSGGRSDEEHNNGTAGAGDSSRVIQEGESGCHIL..........VEPMKLKEHG   549
Consensus

DCR28                  ..........                                                                         257
DCR28-like_rye         EYEDDAIDET                                                                         555
DCR28-like_wheat_B     EYDDAATDET                                                                         555
DCR28-like_wheat_D     DYDDAAIGET                                                                         564
DCR28-like_wheat_A     EYDDDAIDET                                                                         559
Consensus
```

Supplementary Fig. 15. Amino acid alignment between DCR28 and it's A chromosome-paralogs of rye and wheat. Source data are provided as a Source Data file.

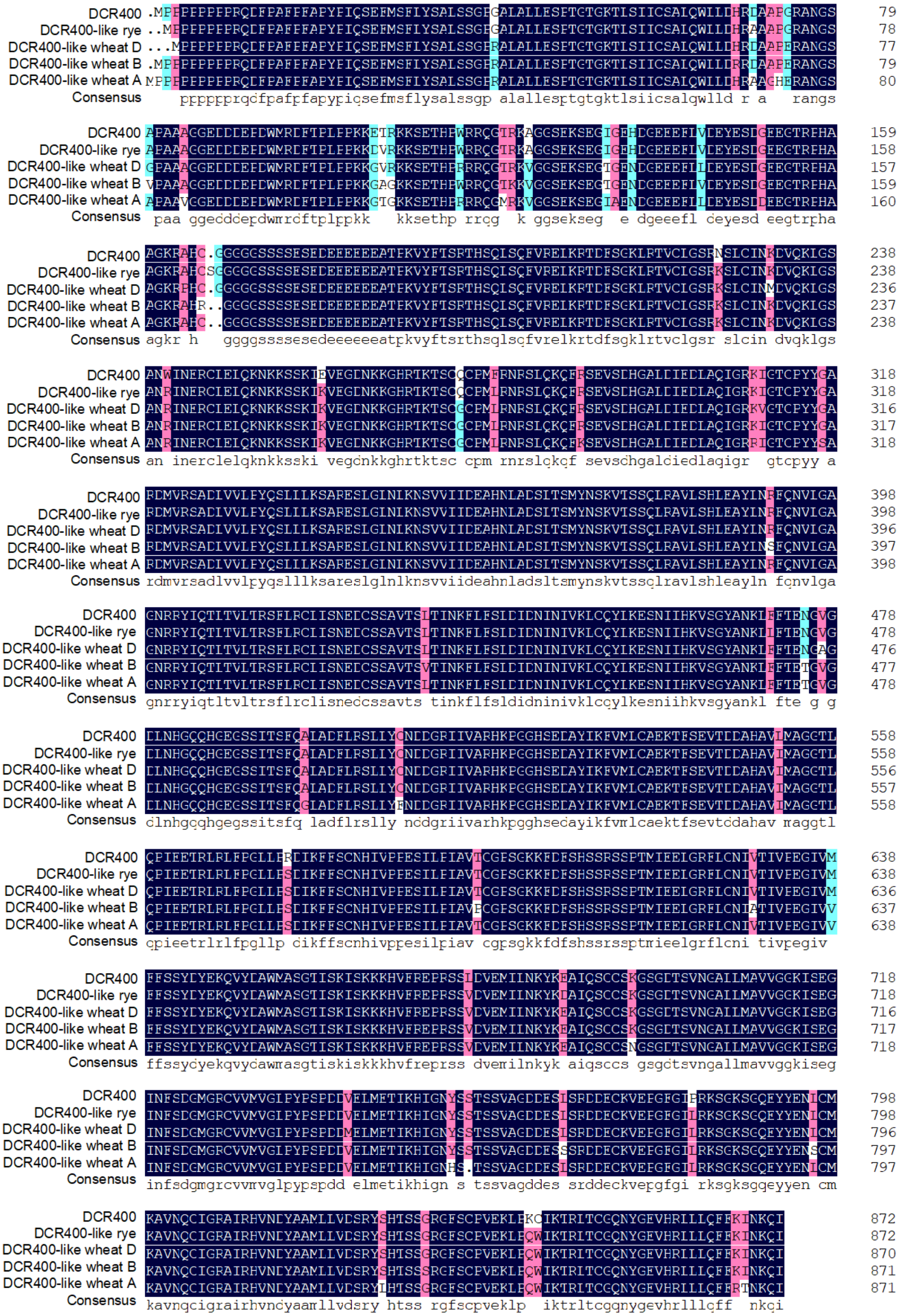

Supplementary Fig. 16. Amino acid alignment between DCR400 and its A chromosome-paralogs of rye and wheat. Source Data are provided as a Source Data file.

Supplementary Fig. 17. Gene tree of Futsch-like proteins calculated with the maximum-likelihood algorithm. The tree was rooted with *Chara braunii* and is based on a conserved module within the protein. The two subclasses of the protein family are indicated as type 1 and type 2. Rye B- and *Ae. speltoides* B-derived DCR28 (arrows) belong to type 2 proteins and group within the Triticeae branch of grasses.

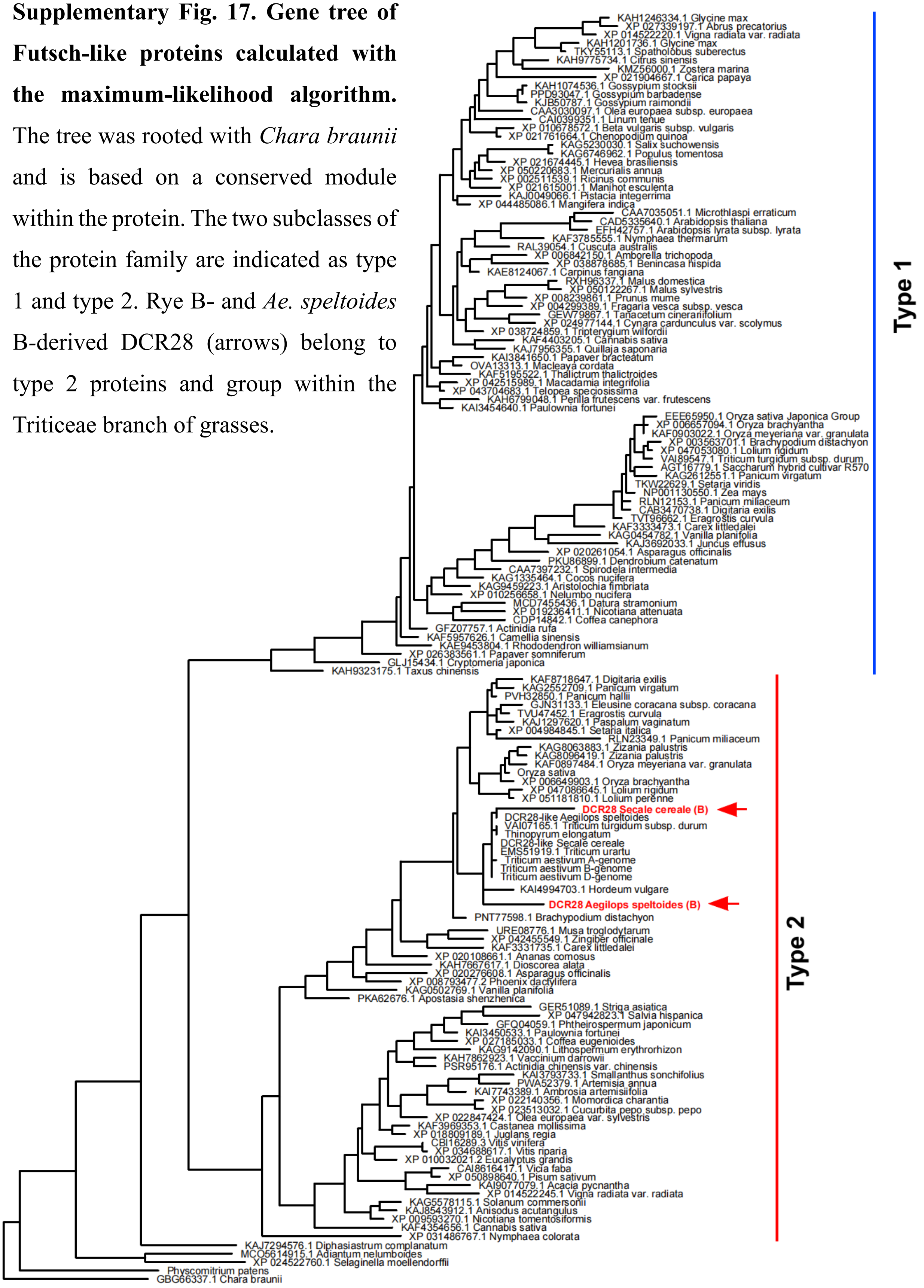

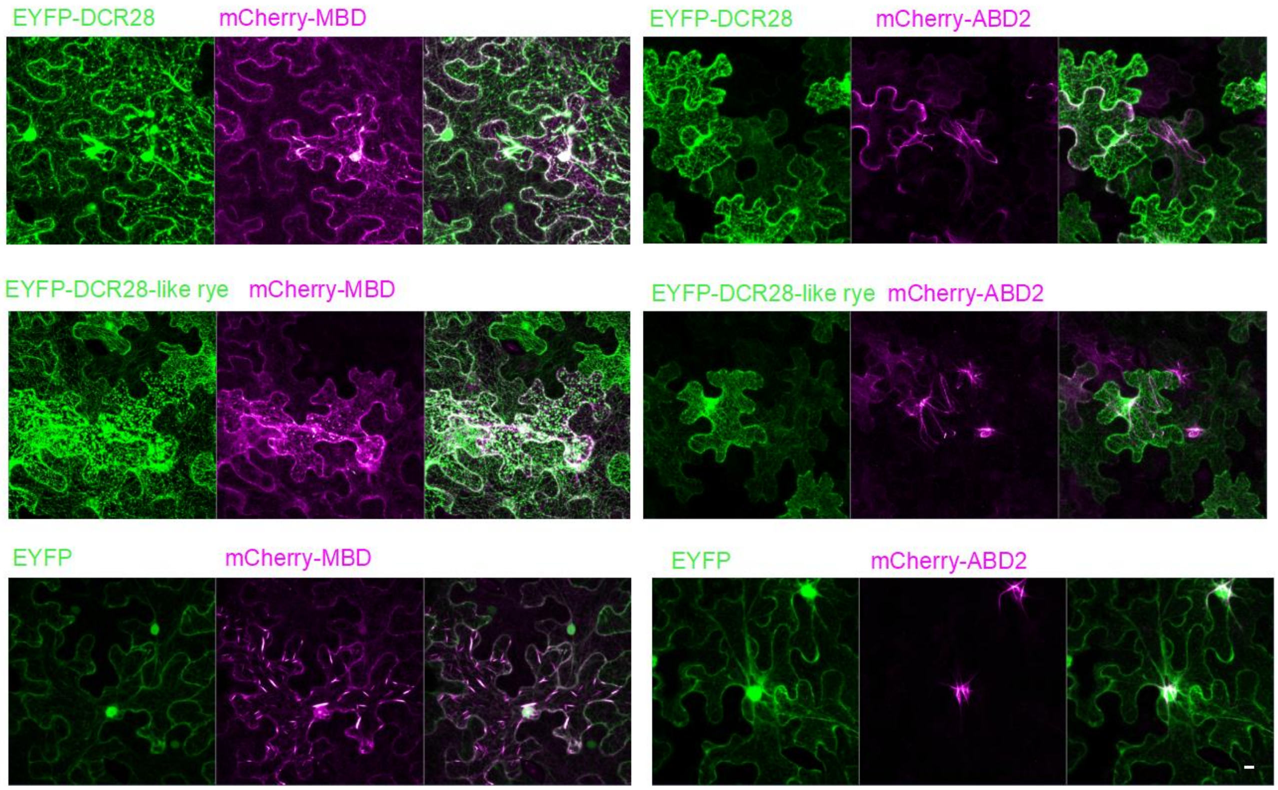

Supplementary Fig. 18. Transient overexpression of EYFP-DCR28, EYFP-DCR28-like rye, and EYFP (all green) together with mCherry-MBD (magenta) and mCherry-ABD2 (magenta), respectively in *N. benthamiana.* Note: colocalization of EYFP-DCR28 and EYFP-DCR28-like rye with the tubulin marker mCherry-MBD. No colocalization exists between EYFP-DCR28 and EYFP-DCR28-like rye with the actin marker mCherry-ABD2. Bar = 10 µm.

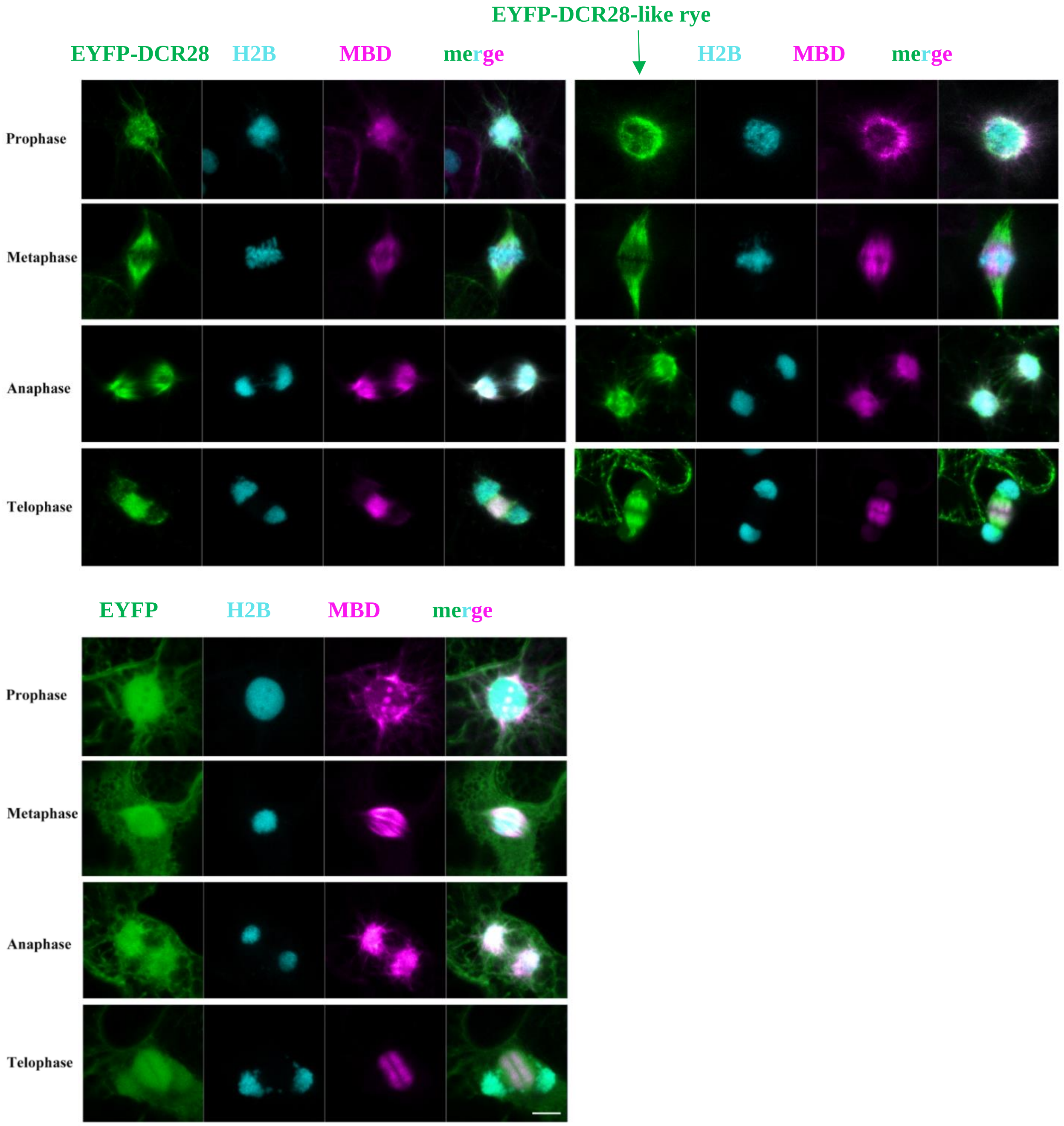

Supplementary Fig. 19. Mitotic cell cycle dynamic of EYFP-DCR28 and EYFP-DCR28-like rye. Transient overexpression of EYFP-DCR28, EYFP-DCR28-like rye, and EYFP (all green) using the cell division-enabled leaf system in the *N. benthamiana* with stable expression of histone H2B-CFP (blue). Transient coexpression mCherry-MBD (magenta) was used as a tubulin-specific marker. Note the spindle-specific distribution of EYFP-DCR28 and EYFP-DCR28-like rye. Bar = 10 µm.

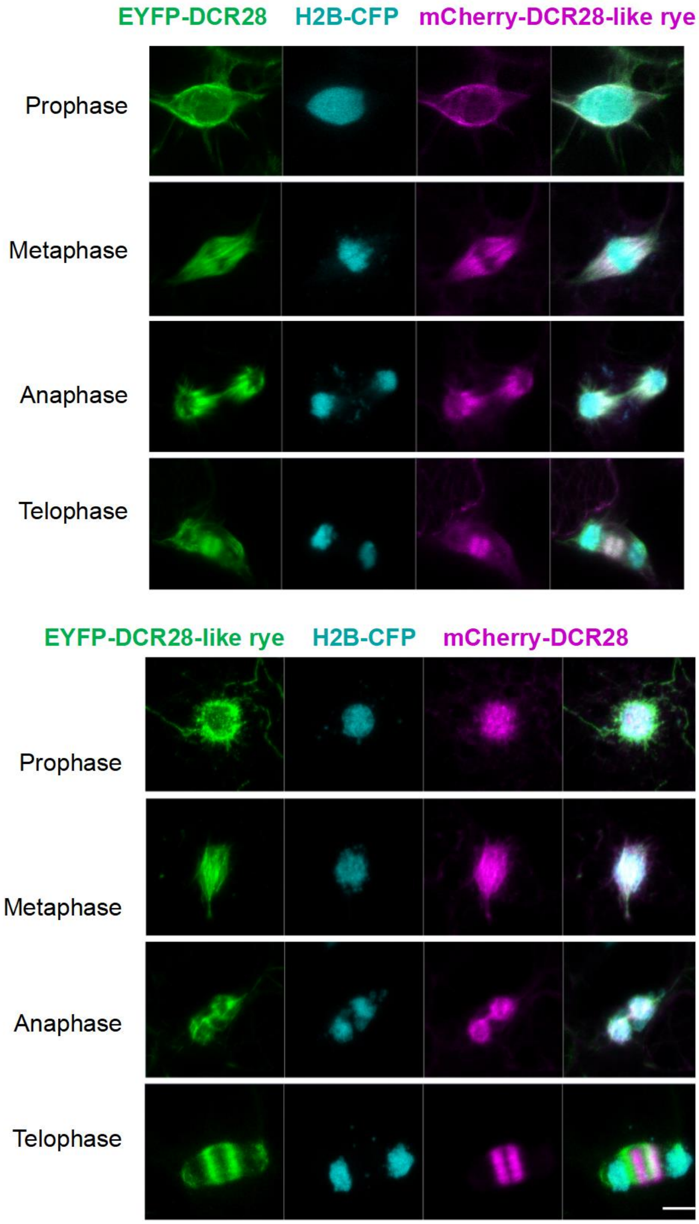

Supplementary Fig. 20. Mitotic cell cycle dynamic and colocaliztion of EYFP-DCR28 and EYFP-DCR28-like rye. Transient co-overexpression of EYFP-DCR28 (green or magenta) and EYFP-DCR28-like rye (magenta or green) using the cell division-enabled leaf system in the *N. benthamiana* with stable expression of histone H2B-CFP (blue). Note the spindle-specific distribution of EYFP-DCR28 and EYFP-DCR28-like rye. Bar = 10 µm.

Supplementary Table 1. Statistics of the primary whole-genome assembly of wheat with rye B chromosomes.

**Statistics of the assembly by QUAST**	
# contigs	2147
# contigs (>= 0 bp)	2147
# contigs (>= 1000 bp)	2145
Largest contig	369738579
Total length	15083438686
Total length (>= 0 bp)	15083438686
Total length (>= 1000 bp)	15083437546
N50	43653268
N75	19499721
L50	85
L75	216
GC (%)	46.13
Mismatches	
# N's	0
# N's per 100 kbp	0
**Statistics of the assembly by BUSCO**	
Complete BUSCOs	3108 (96.0%)
Complete and single-copy BUSCOs	182 (5.6%)
Complete and duplicated BUSCOs	2926 (90.4%)
Fragmented BUSCOs	106 (3.3%)
Missing BUSCOs	22 (0.7%)
Total BUSCO groups searched	3236

Supplementary Table 2. Assignment of scaffolds to wheat chromosomes.

Scaffold	Length (Mb)	Corresponding A chromosome of wheat
1	854.906659	3B
2	820.536733	2B
3	792.189476	2A
4	777.374561	7B
5	762.526675	4A
6	758.879061	3A
7	750.5996	7A
8	746.227681	6B
9	729.085984	5B
10	721.225977	1B
11	719.473869	5A
12	685.02695	4B
13	661.154349	2D
14	655.552324	7D
15	635.678694	3D
16	625.907821	6A
17	599.528823	1A
18	581.431076	5D
19	529.908863	4D
20	507.361415	6D
21	505.391441	1D
Total	14.4 Gb	

Supplementary Table 3. Contigs belongs to the drive control region.

DCR-contigs	Length (bp)
ptg000157l	360262
ptg000534l	503097
ptg000844l	689855
ptg000867l	182053
ptg000879l	68258
ptg000921l	490121
ptg000922l	535399
ptg000937l	160138
ptg000947l	144940
ptg000965l	164745
ptg001057l	89161
ptg001142l	100942
ptg001158l	59870
ptg001501l	53728
ptg002084l	45252
ptg002112l	48517
Total	3.696338 (Mb)

Supplementary Table 4. Oligos and synthesized DNA fragment in this study.

Oligo probe	Sequence
Oligo-D1100-1	5'- FITC-ACCGCATCTCCCTCACTCACAATTTCGATTCCTCCTT-3′
Oligo-D1100-2	5'- FITC-GGTCTCGTTTCCCGCCCAAAGTTTCGCCCC-3′
Oligo-D1100-3	5'- FITC-GTATAGCAAAAGAGTTTCCCAAATAGGCGGCACGA-3′
Oligo-D1100-4	5'- FITC-CGGGTATGGGAACGTAGCATGGAGTTTGGTGG-3′
Grass-5S-1	5′-TAMRA-TCATACCAGCACTAAAGCACCGGATCCCATCAGAAC-3′
Grass-5S-2	5′-TAMRA-GCGTGCTTGGGCGAGAGTAGTACTAGGATGGGTGAC-3′

PCR primer	Left primer (5'-3')	Right primer (5'-3')	Amplicon
Sc9c130	GCATGTCATCGGTAGGATAGG	ACCCCTTCCCTTTCGATCTAC	604 bp
Sc26c38	CAAGACATGCTCACGCTCAG	CGCACTTCCGAGTAACCTGT	521 bp
DCR28-3	TGTCTACGCCGTTTACAATGA	GCTCACGGAATAAAGGGGTCA	947 bp
Bilby	TTTGCGACAATGACTCAAGC	TGTAGCTCATCGTGGAGTCG	582 bp
DCR145-1	CGCAGGTCTCGTGGCTTTAT	AATTGGCAATGTTCCGCTGC	620 bp
DCR154-1	ACAACGGCCAACTCATTCCT	CCTCCCAAGAACTCGTCCAC	790 bp
DCR169-1	TCCTGGATCCATGGACCACT	GAACACCCCTTGAGCACACT	239 bp
DCR260-1	AAGAGGGAATACGGTGCCAG	CAGATCTAGAGTGGGCAGCAG	167 bp
DCR28-2	AGCCGAAAACTCTGCTGGG	GCACGCAAACAAGGTCTCAA	574 bp
DCR398-1	CAAACATTCGGCACCAAGGG	GGCGTAATCTTCTGGAGCGA	742 bp
DCR399-1	TGAGATCCTGCCTCCAACCT	CGGGGAAGGACACGTTCTTT	100 bp
DCR400-2	ATAAGCTGGGTATGGGGCAG	GCGCTGTAGAGGAAGGACAT	399 bp
DCR83-3	TGGCCCTGTCTTCCACTTTC	GGTTTGTCGGGGTCATGAGA	347 bp
AesDCR28-5	CTGACTCTGACTCTGGTGGG	AATGCAAGGCGGAAGAACTC	214 bp
18S rDNA	TGCTACCTGGTTGATCCTGC	ACTAATGCGCCCGGTATTGT	500 bp

Cloning oligo	Sequence (5'-3')
DCR28_5’-UTR	GTCCGC TCCTCCATC TCTTG
DCR28_3’-UTR	GTC TGGTA CTGGCTCA CAAGA
NT-DCR28F	TTGGTC TCTAGGT ATGGA TCCC CGCCC CACC
NT-DCR28R	AAGGTCT CACGAATTA GTCAACT GTATTTGT TGTTGT
EYFP28A2F	TTGGTCTCTCCGCAGAAACCCCGTTCTTTAGCG
EYFP28A2R	AAGGTCTCTCGAACATCTCATCAATGGCATCATCC

Supplementary Table 5. Statistics of the optical mapping.

Optical genome map statistics		
Filtered molecules (MaxInt 2000, >300 kb)	Total length	92.0 Gb
	Molecule N50	428.3 kb
	Molecule coverage*	115 x
Optical map assembly - total	No. of contigs	262
	Total length	411.4 Mb
	Contig N50	5.1 Mb
	Average contig length	1.6 Mb
Optical map assembly – rye B only	No. of contigs	90
	Total length	317.8 Mb
	Contig N50	8.5 Mb
	Average contig length	3.5 Mb

Hybrid scaffold statistics	NGS	NGS involved in HS	HS only	HS + not scaffolded NGS
No. of contigs/scaffolds	176	49	18	181
Total length (Mb)	457.6	371.9	377.1	462.7
Contig/scaffold N50 (Mb)	33.2	29.2	44	38.5
Mean contig/scaffold length (Mb)	2.6	7.6	7.6	2.6
Max contig/scaffold length (Mb)	93	93	98.7	98.7

*Molecule coverage considers 30% contamination of sorted fraction by wheat chromosomes

NGS – input sequence assembly; HS – hybrid scaffolds

Supplementary Table 6. Vectors used in this study.

Name	Backbone vector	Description	Bacteria resistance	Reference/ origin
pICH86966	/	empty vector	Kan	Addgene
pAGM1287-EYFP	pAGM1287	EYFP	Spec	This study
pAGM9121-rbcSE9ter	pAGM9121	terminator	Spec	This study
pICH41258-EYFP-6xGly	pICH41258	*N*-terminal EYFP tag	Spec	This study
pICH41258-mCherry-6xGly	pICH41258	*N*-terminal mCherry tag	Spec	This study
pICH41295:35Sprom	pICH41295	35S promotor	Spec	This study
#1940	pEXPpJOG349_nptII	35S::mCherry-MBD	Spec	This study
#1939	pJOG394	35S::mCherry-ABD2	Spec	This study
CycD3	pGWB2	35S::AtCYCD3;1	Kan	Xu *et al.*[1]
p19	NA	35S:p19	Kan	
pICH86966-35SProm-EYFP	pICH86966	35S::EYFP	Kan	This study
pICH86966-35SProm-EYFP-DCR28	pICH86966	35S::EYFP-DCR28	Kan	This study
pICH86966-35SProm-mCherry-DCR28	pICH86966	35S:: mCherry-DCR28	Kan	This study
pICH86966-35SProm-EYFP-DCR28A	pICH86966	35S::EYFP-DCR28A	Kan	This study
pICH86966-35SProm-mCherry-DCR28A	pICH86966	35S::mCherry-DCR28A	Kan	This study

Bacteria selection resistance = resistance used for selection; Spec = Spectinomycin, Kan = Kanamycin

Supplementary Table 7. The information on the whole-genome sequencing data (PRJEB69479).

PacBio HiFi	Plant material	Sequencer	Bases (Gb)	Median read length (bp)/ Number of reads
1st library	wheat cv. Chinese Spring with 6 rye B chromosomes	Sequel IIe	91.59	20891/ 4322100
2nd library	wheat cv. Chinese Spring with 6 rye B chromosomes	Revio	64.66	12695/ 5026842
3rd library	wheat cv. Chinese Spring with 6 rye B chromosomes	Revio	101.52	15485/ 6381967
**ONT ultra-long**			**Bases (Gb) pass Q10**	**Median read length (bp)/ Number of reads**
1st library	wheat cv. Chinese Spring with 6 rye B chromosomes	PromethION	35.91	64.59/ 2.1 M
2nd library	wheat cv. Chinese Spring with 6 rye B chromosomes	PromethION	33.09	44.97/ 3.46 M
**Hi-C**			**Total base**	**Filtered base**
Library 1	wheat cv. Chinese Spring with 2 rye B chromosomes	NovaSeq6000	22,001,537,99	16831066437
Library 2	wheat cv. Chinese Spring with 2 rye B chromosomes	NovaSeq6000	21282053240	16294486947
Library 3	wheat cv. Chinese Spring with 2 rye B chromosomes	NovaSeq6000	38491554630	29251047623
Library 4	wheat cv. Chinese Spring with 2 rye B chromosomes	NovaSeq6000	38829854918	29586496204
Library 5	wheat cv. Chinese Spring with 2 rye B chromosomes	NovaSeq6000	36697726973	27836665038
Library 6	wheat cv. Chinese Spring with 2 rye B chromosomes	NovaSeq6000	37529776779	28490635429
**Short read sequencing**			**Read length**	**Clean base**
Library 1	wheat cv. Chinese Spring with 2 rye B chromosomes	DNBSEQ (BGI)	PE150	36,040,959,900
Library 2	wheat cv. Chinese Spring with 2 rye B variant B^k-3	DNBSEQ (BGI)	PE150	36,007,339,500
Library 3	wheat cv. Chinese Spring with 3 rye B variant B^k-2	DNBSEQ (BGI)	PE150	36,003,318,600

Supplementary reference

1 Xu, J., Lee, Y. R. J. & Liu, B. Establishment of a mitotic model system by transient expression of the D-type cyclin in differentiated leaf cells of tobacco (*Nicotiana benthamiana*). *New Phytol* **226**, 1213-1220 (2020).
