# Supplementary material for: The genetic mechanism of B chromosome drive in rye illuminated by chromosome-scale assembly
Source: Nat Commun. 2024 Nov 8;15:9686. doi: 10.1038/s41467-024-53799-w (PMC11549084; doi:10.1038/s41467-024-53799-w)
Supplement: Supplementary file 3 — Description of Additional Supplementary Files [file 41467_2024_53799_MOESM3_ESM.pdf]

Description of Additional Supplementary Files

Supplementary Movie 1. Pollen-FISH combined with super-resolution microscopy (3D SIM) was used to determine the position of two rye drive-positive B chromosomes in mature pollen of wheat. Repeat D1100 (green) indicates the B chromosome and 5S rDNA (magenta) is A chromosome-specific marker used as a positive FISH control. The nuclei were stained by DAPI (blue).

Supplementary Data 1. The HiFi reads coverage of the contigs from the whole-genome assembly of wheat with rye B chromosomes.

Supplementary Data 2. Utilization of Hi-C and optical mapping data to link the contigs into super-scaffolds.

Supplementary Data 3. The repeat distribution on the contigs from whole-genome assembly of wheat with rye B chromosomes (1-Mb windows).

Supplementary Data 4. The final organization of contigs in pseudomolecule.

Supplementary Data 5. Description of RNA-seq samples.

Supplementary Data 6. Annotation of the genes on the rye B chromosome. The location, coding ability, homologous gene on the A chromosomes of the genes on the rye B chromosome. Primary confidence class are used to indicate the level of confidence or reliability in the Rye cv. Lo7 annotation. HC: High Confidence, LC: Low Confidence.

Supplementary Data 7. A subtractive approach reveals common DEGs and their PCR results. A subtractive approach reveals common DEGs and the summary of the genomic PCR (see Supplementary Fig. 8) to test the rye B drive control region-specific location of preselected candidate sequences. Genomic DNA of wheat with B variants possessing either a drive-functional drive control region ($2B^s$, $2B^k$, $1B^k$-2) or nonfunctional drive control region ($1B^s$-8, $1B^k$-1, $2B^k$-3) was used as a PCR template.

Supplementary Data 8. The expression level of the rye B-encoded genes during the first pollen mitosis (PMI). Expression value: transcript per million (TPM).

Supplementary Data 9. The expression level of the five candidates in different tissues.

Supplementary Data 10. The sequence of the de novo assembled transcript and its translated protein of AesDCR28.

Supplementary Data 11. The sequence of synthesized DNA fragment DCR28A1_opt.
