# Supplementary material for: The genetic mechanism of B chromosome drive in rye illuminated by chromosome-scale assembly
Source: Nat Commun. 2024 Nov 8;15:9686. doi: 10.1038/s41467-024-53799-w (PMC11549084; doi:10.1038/s41467-024-53799-w)
Supplement: Supplementary file 16 — Reporting Summary [file 41467_2024_53799_MOESM16_ESM.pdf]

nature portfolio

Corresponding author(s): Andreas Houben
Jianyong Chen

Last updated by author(s): 15/10/24

Reporting Summary

Nature Portfolio wishes to improve the reproducibility of the work that we publish. This form provides structure for consistency and transparency in reporting. For further information on Nature Portfolio policies, see our Editorial Policies and the Editorial Policy Checklist.

Statistics

For all statistical analyses, confirm that the following items are present in the figure legend, table legend, main text, or Methods section.

n/a	Confirmed	
☐	☒	The exact sample size (*n*) for each experimental group/condition, given as a discrete number and unit of measurement
☐	☒	A statement on whether measurements were taken from distinct samples or whether the same sample was measured repeatedly
☐	☒	The statistical test(s) used AND whether they are one- or two-sided<br>*Only common tests should be described solely by name; describe more complex techniques in the Methods section.*
☒	☐	A description of all covariates tested
☒	☐	A description of any assumptions or corrections, such as tests of normality and adjustment for multiple comparisons
☒	☐	A full description of the statistical parameters including central tendency (e.g. means) or other basic estimates (e.g. regression coefficient) AND variation (e.g. standard deviation) or associated estimates of uncertainty (e.g. confidence intervals)
☐	☒	For null hypothesis testing, the test statistic (e.g. *F*, *t*, *r*) with confidence intervals, effect sizes, degrees of freedom and *P* value noted<br>*Give P values as exact values whenever suitable.*
☒	☐	For Bayesian analysis, information on the choice of priors and Markov chain Monte Carlo settings
☐	☒	For hierarchical and complex designs, identification of the appropriate level for tests and full reporting of outcomes
☒	☐	Estimates of effect sizes (e.g. Cohen's *d*, Pearson's *r*), indicating how they were calculated

Our web collection on statistics for biologists contains articles on many of the points above.

Software and code

Policy information about availability of computer code

Data collection	Microscope images were taken using an Olympus BX61 fluorescence microscope equipped with an ORCA-ER CCD camera (Hamamatsu) and a deconvolution system. High- resolution images were taken using a 63x/1.40 objective of an Elyra PS.1 super-resolution microscope system and the software ZENBlack (Carl Zeiss GmbH). Image stacks were captured separately for each fluorochrome using 561, 488, and 405 nm laser lines for excitation and appropriate emission filters. Short-read sequencing data were generated by Illumina HiSeq 2500 device (paired-end, 2 x101 cycles and DNBSEQ sequencing platform. HiFi CCS Sequencing was performed using the Pacific Biosciences Sequel IIe device and Pacific Biosciences Revio platform. Nanopore sequencing was performed PromethION sequencer. Hi-C sequencing was performed using the NovaSeq6000 device. Optical genome mapping data was generated by the Bionano Genomics Saphyr platform. The protein sequence of DCR28 was aligned to PANTHER (http://www.pantherdb.org/) and used in a BLASTp search in the NCBI non-redundant (nr) protein sequence database to identify DCR28-like proteins.
Data analysis	Images were analyzed using the cellSens Dimension software (Olympus, v1.11) and Adobe Photoshop (v13.0). The 3D -image stacks were used to generate Suppl. Movie 1 using the Imaris 9.7 (Bitplane) software. Bionano Solve software (version 3.6.1_11162020; parameters "optArguments_nonhaplotype_noES_noCut_DLE1_saphyr.xml") was used to de novo assembley of an optical genome map (OGM). hifiasm (v0.19.3-r572; parameters: -l 0 -D 20, Ultra-long ONT integration) was used for genome assembly. The coverage of HiFi reads on each contig was extracted from the Graphical Fragment Assembly (GFA) file output by hifiasm. Contig statistics were calculated with Quast (v2.3) and gene content completeness was evaluated with Benchmarking Universal Single-Copy Orthologs (BUSCO) (v4.1.2; dataset: Viridiplantae Odb10) . The Arima Genomics mapping pipeline (https://github.com/ArimaGenomics/mapping_pipeline) was used to process the Hi-C data, including read mapping to the contigs, read filtering, read pairing, and PCR duplicate removal, and scaffolding was performed using YaHS (v1.2a.2). Hi-C contact maps were generated using Juicebox (https://github.com/aidenlab/Juicebox). blastn (blast program: megablast, v2.10.1) was used for alignment and synteny analysis and the results were visualized by Dot plot using R (v4.01). Bionano Solve (version 3.6.1.11162020) was used for automated hybrid scaffolding (HS) of optical mapping data. fasta file was generated with agptools (https://

warrenlab.github.io/agptools/). pyGenomeTracks (v3.8) was used for visualizing data on the pseudomolecule. The alignments were extracted via samtools (v1.9). Bowtie2 (v 2.5.0, default) was used for short-read mapping. HISAT2 (v 2.2.1, default parameters), StringTie (v 2.1.1, default parameters) was used for mapping-annotation of the RNA-seq data. gffread (Version 0.12.6) was used for generating the transcripts sequences. TransDecoder (v 5.5.0) was used to annotate coding regions within transcripts. Differential expression analysis was performed using Salmon (v.3.0, default parameters)- DESeq2 (v 1.34.0). CD-HIT-EST (similarity threshold: 0.8, v1.3) was used for clustering the differentially expressed genes. The PANTHER (Protein Analysis Through Evolutionary Relationships) classification system (http://www.pantherdb.org) was used to infer the likely functional roles of the translated proteins. ClustalX (v2.1) was used for multiple sequence alignment.

For manuscripts utilizing custom algorithms or software that are central to the research but not yet described in published literature, software must be made available to editors and reviewers. We strongly encourage code deposition in a community repository (e.g. GitHub). See the Nature Portfolio guidelines for submitting code & software for further information.

Data

Policy information about availability of data

All manuscripts must include a data availability statement. This statement should provide the following information, where applicable:

- Accession codes, unique identifiers, or web links for publicly available datasets
- A description of any restrictions on data availability
- For clinical datasets or third party data, please ensure that the statement adheres to our policy

Raw sequence reads (Whole-genome sequencing short reads, PacBio, Nanopore, Hi-C sequencing, optical mapping) are available from the European Nucleotide Archive (ENA) under accession numbers PRJEB69479. RNA-seq is under PRJEB46034. B chromosome assembly is GCA_964027155 under the study of PRJEB69479.

Research involving human participants, their data, or biological material

Policy information about studies with human participants or human data. See also policy information about sex, gender (identity/presentation), and sexual orientation and race, ethnicity and racism.

Reporting on sex and gender	N/A
Reporting on race, ethnicity, or other socially relevant groupings	N/A
Population characteristics	N/A
Recruitment	N/A
Ethics oversight	N/A

Note that full information on the approval of the study protocol must also be provided in the manuscript.

Field-specific reporting

Please select the one below that is the best fit for your research. If you are not sure, read the appropriate sections before making your selection.

☒ Life sciences ☐ Behavioural & social sciences ☐ Ecological, evolutionary & environmental sciences

For a reference copy of the document with all sections, see nature.com/documents/nr-reporting-summary-flat.pdf

Life sciences study design

All studies must disclose on these points even when the disclosure is negative.

Sample size	We decided the sample size based on available literatures in the field, our own experience in previous studies, and requirement for corresponding protocols. The sample sizes used for all experiments provided sufficient resolving power.
Data exclusions	No data was excluded from the analysis.
Replication	The number of replication are indicated in the section Methods. For fluorescence in situ hybridization, at least two independent experiments were carried out to confirm the reproducibility of the labeling patterns. All replications were successful.
Randomization	A randomization is not relevant for this study because no treatment swere compared with each other. Hower, the plants fo used for RNAseq analysis were grown under the same condition in a greenhouse. Only for genome assembly, we used the DNA of a single genotpye for long-read sequencing, to avoid problems due to the highly heterozygous genome of the wheat rye B addition line,
Blinding	All the experiments were performed without prior knowledge of the final outcome, and therefore blinding was not applied.

Reporting for specific materials, systems and methods

We require information from authors about some types of materials, experimental systems and methods used in many studies. Here, indicate whether each material, system or method listed is relevant to your study. If you are not sure if a list item applies to your research, read the appropriate section before selecting a response.

Materials & experimental systems

n/a	Involved in the study
☒	☐ Antibodies
☒	☐ Eukaryotic cell lines
☒	☐ Palaeontology and archaeology
☒	☐ Animals and other organisms
☒	☐ Clinical data
☒	☐ Dual use research of concern
☐	☒ Plants

Methods

n/a	Involved in the study
☒	☐ ChIP-seq
☒	☐ Flow cytometry
☒	☐ MRI-based neuroimaging

Dual use research of concern

Policy information about dual use research of concern

Hazards

Could the accidental, deliberate or reckless misuse of agents or technologies generated in the work, or the application of information presented in the manuscript, pose a threat to:

No	Yes
☒	☐ Public health
☒	☐ National security
☒	☐ Crops and/or livestock
☒	☐ Ecosystems
☒	☐ Any other significant area

Experiments of concern

Does the work involve any of these experiments of concern:

No	Yes
☒	☐ Demonstrate how to render a vaccine ineffective
☒	☐ Confer resistance to therapeutically useful antibiotics or antiviral agents
☒	☐ Enhance the virulence of a pathogen or render a nonpathogen virulent
☒	☐ Increase transmissibility of a pathogen
☒	☐ Alter the host range of a pathogen
☒	☐ Enable evasion of diagnostic/detection modalities
☒	☐ Enable the weaponization of a biological agent or toxin
☒	☐ Any other potentially harmful combination of experiments and agents

Plants

Seed stocks

Cultivated rye (Secale cereale L. subsp. cereale) of the Japanese JNK strain without and with additional standard or deficient rye Bs (defB), which lost the ability to drive due to the loss of the nondisjunction control region and weedy rye containing B chromosomes collected from Pakistan (Secale cereale L. subsp. segetale, no. 34) and Afghanistan (Secale cereale L. subsp. afghanicum). Wheat (Triticum aestivum L., cv Chinese Spring) without and with different variants of rye B chromosome (Bs, Bs-8, BR, Bk-1, Bk-2 15, and Bk-3). Wild type and transgenic Nicotiana benthamiana expressing the reporter construct 35S::CFP-histone H2B. Aegilops speltoides Tausch with or without B chromosomes from Tartus, Syria (K2 and D2), Katzir, Israel (H7), Ramat Hanadiv, Israel (P12) were grown under long-day conditions of 16 h light at 25 °C and 8 h dark at 16°C, 40% relative humidity, and 50-60 kilolux light intensity. They were vernalized at 4 °C for four weeks at the third leaf stage and then moved back to the greenhouse.

Novel plant genotypes

The Bk-3 was identified when we screened the selfing progenies of wheat with Bk-2 by FISH, it lost the ability of drive.

Authentication

Fluorescence in situ hybridization using B chromosome-specific probes was used to confirm each genotype.
